# A Simple Experimental Approach to Understanding the Formation of Advanced Glycation End Products

**DOI:** 10.7759/cureus.84871

**Published:** 2025-05-27

**Authors:** Kaho Miyazaki, Kumiko Miyazaki, Kuniyoshi Kaseda

**Affiliations:** 1 Health Sciences, Oita Hofu Junior High School, Oita, JPN; 2 Research and Development, Uchi-Lab, Beppu, JPN; 3 Research and Development, Imamura Chemical Termite Control Co. Ltd., Oita, JPN

**Keywords:** advanced glycation end products, ages, collagen, colorimetric sheet, glycation, healthy aging, healthy life expectancy

## Abstract

Introduction: Advanced glycation end products (AGEs) are non-enzymatically formed through reactions between sugars and proteins. Over the past decades, accumulating evidence has linked AGEs to the acceleration of aging and various pathological processes. Despite their biological significance, AGEs remain largely unrecognized by the general public. Therefore, new initiatives are needed to raise awareness of AGEs.

Methods: An AGE colorimetric sheet was developed. The glycation process of collagen with saccharides was analyzed using the sheet under various conditions, along with the physical properties of the protein.

Results: The glycation process of collagen depended on time, dose, and temperature. Additionally, we identified a correlation between AGE levels and the mechanical toughness of the collagen matrix. Furthermore, commercially available beverages containing fructose significantly accelerated collagen glycation.

Conclusion: Our data demonstrate that the novel colorimetric sheet is a practical tool for assessing AGE formation without requiring specialized analytical instruments. This experimental system can enhance public health awareness, contributing to advances in preventive medicine and promoting healthy aging.

## Introduction

The Maillard reaction was first reported in 1912 by Louis-Camille Maillard. Approximately a century later, in the mid-1990s, attention was paid to endogenous protein glycation. Since then, research in this field has dramatically accelerated [1,2]. It has been demonstrated that the accumulation of advanced glycation end products (AGEs) in the body is implicated not only in diabetes complications but also in aging-related conditions such as pigmentation, wrinkles, and joint pain, as well as in myocardial infarction, Alzheimer’s disease, and osteoporosis, among others [3-6]. Today, the glycation level of hemoglobin A1c in the blood serves as a key indicator of blood sugar control, playing a crucial role in diabetes management and lifestyle disease prevention [7,8].

When reducing sugars bind non-enzymatically to the amino groups of proteins, lipids, or nucleic acids, they initially form a Schiff base. Via the Amadori rearrangement, these intermediates ultimately contribute to the formation of AGEs. The final stage of this process is irreversible, indicating that once AGEs are formed, the original protein function cannot be restored. The half-life of AGEs ranges from several years to decades, contributing to protein cross-linking both within and between molecules, which further leads to the loss of function in proteins and tissues. Additionally, AGEs bind to their receptors, triggering a cascade that enhances oxidative stress and inflammation. This cascade ultimately contributes to insulin resistance, diabetic vascular complications, and other metabolic disorders [9-12].

As developed countries face a rapidly aging society, increasing emphasis is placed on extending health span and advancing preventive medicine. However, public awareness of glycation and AGEs remains low, as AGE research requires specialized knowledge and expensive equipment, making it inaccessible to non-experts [13]. To address this gap, we developed a simple and cost-effective method for the visualization and quantification of AGE levels, with the goal of enhancing public understanding and awareness of AGEs, particularly in educational environments, healthcare contexts, and community outreach initiatives.

## Materials and methods

Development of the AGE colorimetric sheet

The surface reflectance of each sample color was measured at least three times using a spectrocolorimeter (SD6000, Nippon Denshoku Industries, Co., Ltd., Tokyo, Japan). The averaged K/S values (specular component exclude (SCE)) at 450 nm were calculated as an indicator of color intensity using the software ColorMate Pro Ver. 1.08.02 (Nippon Denshoku Industries, Co., Ltd., Tokyo, Japan). The analysis was based on the Kubelka-Munk model: K/S = (1−R)²/2S, where K is the absorption coefficient, S is the scattering coefficient, and R is the surface reflectance [14,15]. By assigning a value of 0 to the initial color of the reactants and 100 to the final color, we calculated the relative K/S values for each color. As a result, an AGE colorimetric sheet was prepared to visually represent the AGE levels in the glycosylated reactants.

Analysis of glycation reactions

Gelatin (Morinaga & Co., Ltd., Tokyo, Japan) was thoroughly dissolved in tap water at 80°C. The solution was incubated in the presence or absence of saccharides. Sucrose (Mitsui DM Sugar Co., Ltd., Tokyo, Japan), glucose (S-ING Co., Ltd., Sapporo, Japan), and fructose (Wellneo Sugar Co., Ltd., Tokyo, Japan) were used. The temperature was maintained at 40°C or 60°C using a yogurt maker (model IYM-014, Iris Ohyama Inc., Sendai, Japan). The incubation duration for each experiment is provided in the respective figure legends. The color of each reactant was compared to that of the AGE colorimetric sheet to identify the glycation level. The experiments were carefully conducted under various sugar doses, incubation times, and temperatures to reinforce their reliability. The Mann-Kendall test was used to assess whether a statistically significant upward trend exists in the data series. A UV transilluminator (SLB-01W, MaestroGen Inc., Hsinchu, Taiwan) was used to visualize AGE-derived fluorescence. The experiments were performed in duplicate.

Examination of the physical properties of glycated collagen

After incubating gelatin sol in the presence or absence of the saccharide at 60°C for three days, the solution was cooled to 6°C to allow collagen to solidify. Three hours later, the test containers were tilted to examine the gelation state of the solution. Additionally, to investigate the mechanical toughness of collagen gel, after cooling the collagen solutions for 12 hours, the gels were returned to room temperature and placed on a 15° incline. After 10 minutes, the distance from the bottom of the containers to the leading edge of the gel was measured. The experiments were repeated three to five times under similar conditions. Representative data are presented.

Analysis of the glycation potential of commercial beverages

Commercially available beverages were used as a substitute for water in preparing collagen solutions. The beverages included unsweetened carbonated water (Sparkling Water, Japan Foods Co., Ltd, LTD., Chiba, Japan), sweetened water (I LOHAS, Coca-Cola (Japan) Company, Limited, Tokyo, Japan), and sweetened carbonated water (Mitsuya Cider, Asahi Soft Drinks Co., Ltd., Tokyo, Japan). After incubating each collagen solution at 60°C, the color of each reactant was compared to that of the AGE colorimetric sheet to identify the AGE level. The experiments were performed in duplicate or more times. Representative data are presented.

Analysis of sugar content

Sugar content was measured using a saccharimeter (Brix 0-55%, FN018A, Yu-An Co., Ltd., Okinawa, Japan).

## Results

Development of the AGE colorimetric sheet

To observe glycation reactions, gelatin was used. Gelatin is primarily composed of collagen, which accounts for approximately 30% of the total proteins in the human body and plays a crucial role in the skin, joints, blood vessels, bones, cornea, and many other tissues, making it an essential factor in aging and disease research. Additionally, gelatin solutions are transparent and inexpensive. Commonly available saccharides, including sucrose, glucose, and fructose, were used.

**Figure 1:**
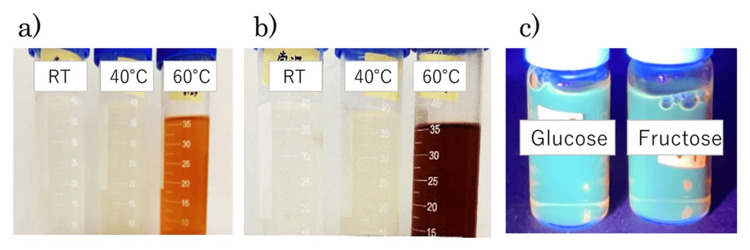
AGE formation induced by fructose (a) Early-stage glycation on day 45 at room temperature (left), 40°C (middle), and 60°C. (b) The color of the solution becomes darker on day 58. (c) Fluorescence emission observed when browned collagen solution was exposed to ultraviolet light (left: glucose-glycated collagen, right: fructose-glycated collagen) AGE: advanced glycation end products

The color of gelatin-containing solutions in the presence of saccharides was recorded over a period of six months at room temperature, 40°C, and 60°C. As the glycation reaction progressed, the transparent solution gradually turned pale yellow, then deepened in color to reddish-brown, and finally dark brown (Figures 1a, 1b). Accordingly, a time-dependent increase in optical density (400-500 nm) was observed (data not shown). Furthermore, when the glycated solution was exposed to ultraviolet light, it emitted distinct fluorescence, confirming AGE formation (Figure 1c).

**Figure 2:**
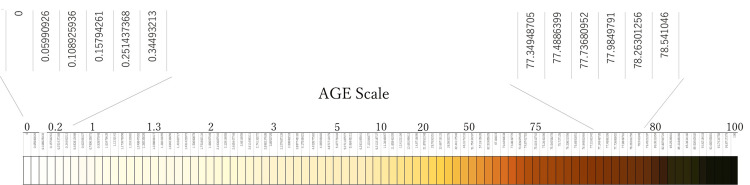
AGE colorimetric sheet The AGE colorimetric sheet was developed based on the Kubelka-Munk model (wavelength 450 nm, SCE). The relative K/S value indicates the glycation level (0 to 100). K/S = (1−R)²/2S, where K = absorption coefficient, S = scattering coefficient, and R = surface reflectance. Enlarged numbers in the figure represent the original K/S values. AGE: advanced glycation end products; SCE: specular component exclude

Under the conditions tested in this study, a similar time-dependent color change was observed. These experiments were repeatedly conducted to record subtle differences in color. Ninety collected colors were arranged in chronological order. The surface reflectance of each color was measured with a spectrocolorimeter. The mean K/S value at 450 nm, an indicator of color intensity based on the Kubelka-Munk model, was calculated. By defining the initial stage of glycation as 0 and the final stage as 100, the relative values for each color were derived to complete an AGE colorimetric sheet, as shown in Figure 2.

Analysis of collagen glycation reactions

**Figure 3:**
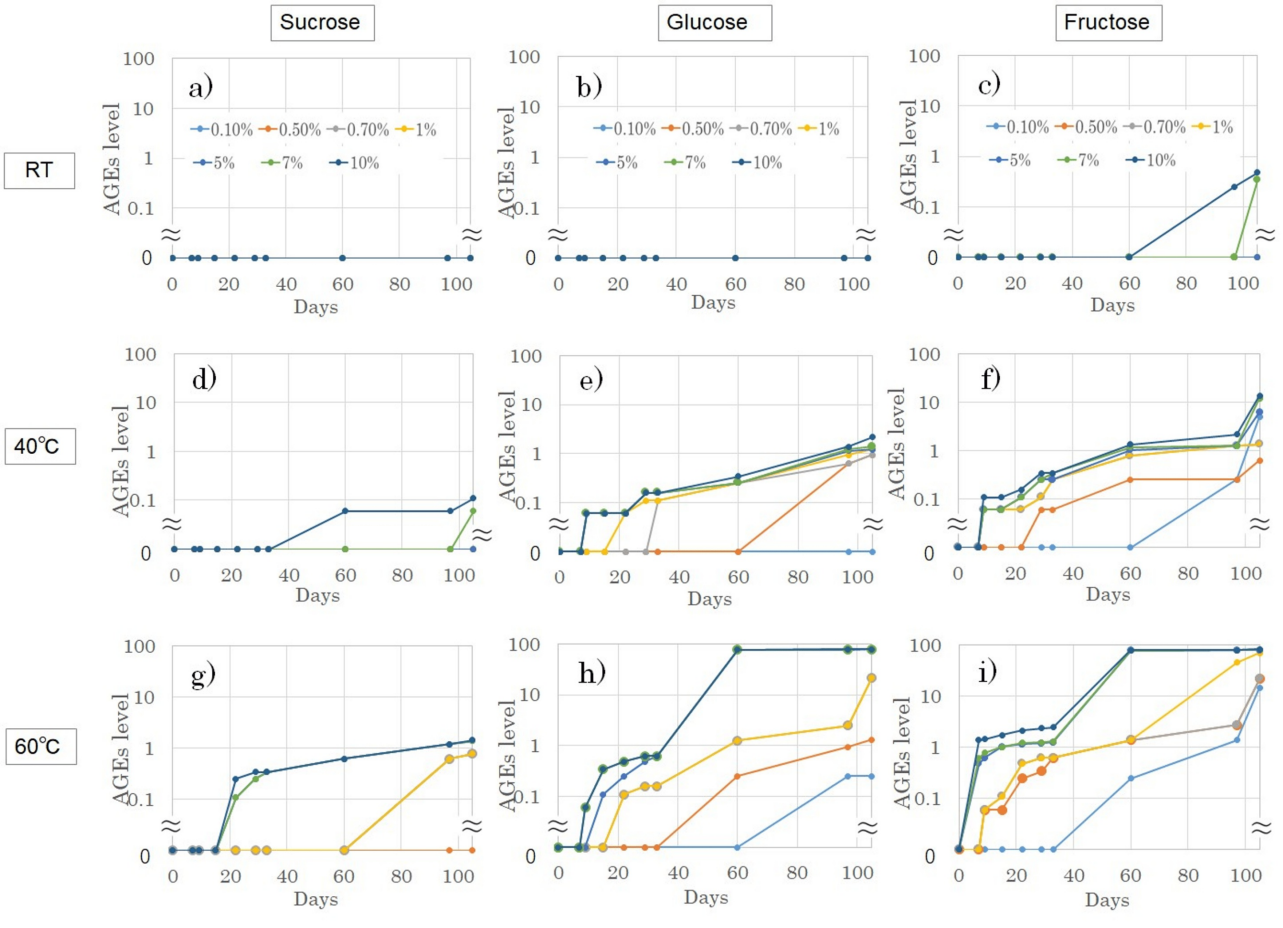
Analysis of glycation reactions using the AGE colorimetric sheet Collagen solutions with different sugar concentrations were incubated for 105 days at room temperature (a–c), 40°C (d–f), and 60°C (g–i). Sucrose (a, d, g), glucose (b, e, h), and fructose (c, f, i) at concentrations of 0.1% (light blue), 0.5% (orange), 0.7% (gray), 1% (yellow), 5% (blue), 7% (green), and 10% (navy) were tested. Data points may not be visible when the values are 0. AGE: advanced glycation end products

Gelatin solutions were incubated with sucrose, glucose, or fructose at various temperatures, and the glycation levels were evaluated using the AGE colorimetric sheet. The glycation levels were found to depend on incubation time, sugar concentration, and temperature (Figure 3). 

Additionally, another experiment revealed that glycation levels depend on collagen concentration (data not shown). These findings confirm that collagen glycation follows the general principles of chemical reactions, being influenced by reactant concentration, reaction temperature, and time. Among the tested saccharides, sucrose exhibited minimal activity in collagen glycation, whereas glucose and fructose promoted glycation more efficiently, indicating that reducing sugars are involved in the reaction [16]. Notably, fructose exhibited the highest glycation potential, which is consistent with previous findings [17-19].

Physical strength of glycated collagen

**Figure 4:**
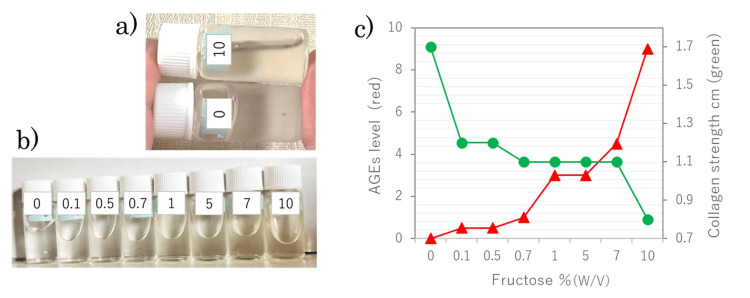
Physicochemical properties of collagen altered by glycation (a) Gelatin was incubated with or without 10% fructose at 60°C for three days. After cooling at 6°C for three hours, the containers were tilted. (b) Gelatin glycated with different fructose concentrations was left at 6°C for 12 hours, then returned to room temperature and placed on a 15° incline for 10 minutes. (c) Glycation levels were assessed using the AGE colorimetric sheet (red), and the gelation index of collagen was measured as the distance from the container's bottom to the top edge of the gelled gelatin (green). AGE: advanced glycation end products

After reacting gelatin with fructose at 60°C for three days, the solution was kept at 6°C for three hours. When the test container was tilted, the gelatin without fructose formed a solid gel, whereas the gelatin glycated with fructose remained in a sol state (Figure 4a). Figure 4b shows the results of gelatin glycation with different concentrations of fructose. After the glycation reaction, the solution was kept at 6°C for 12 hours. Then, when the tubes were left on a 15° incline for 10 minutes at room temperature, the effects of fructose became evident. First, as the fructose concentration increased, the yellow coloration became more pronounced, which was consistently quantified with the AGE colorimetric sheet. Figure 4c illustrates the relationship between the glycation level and the distance from the container bottom to the top edge of the gelled gelatin. The figure shows that collagen gel becomes more likely to remain in a sol state as the glycation reaction progresses.

Glycation potential of commercial beverages

**Figure 5:**
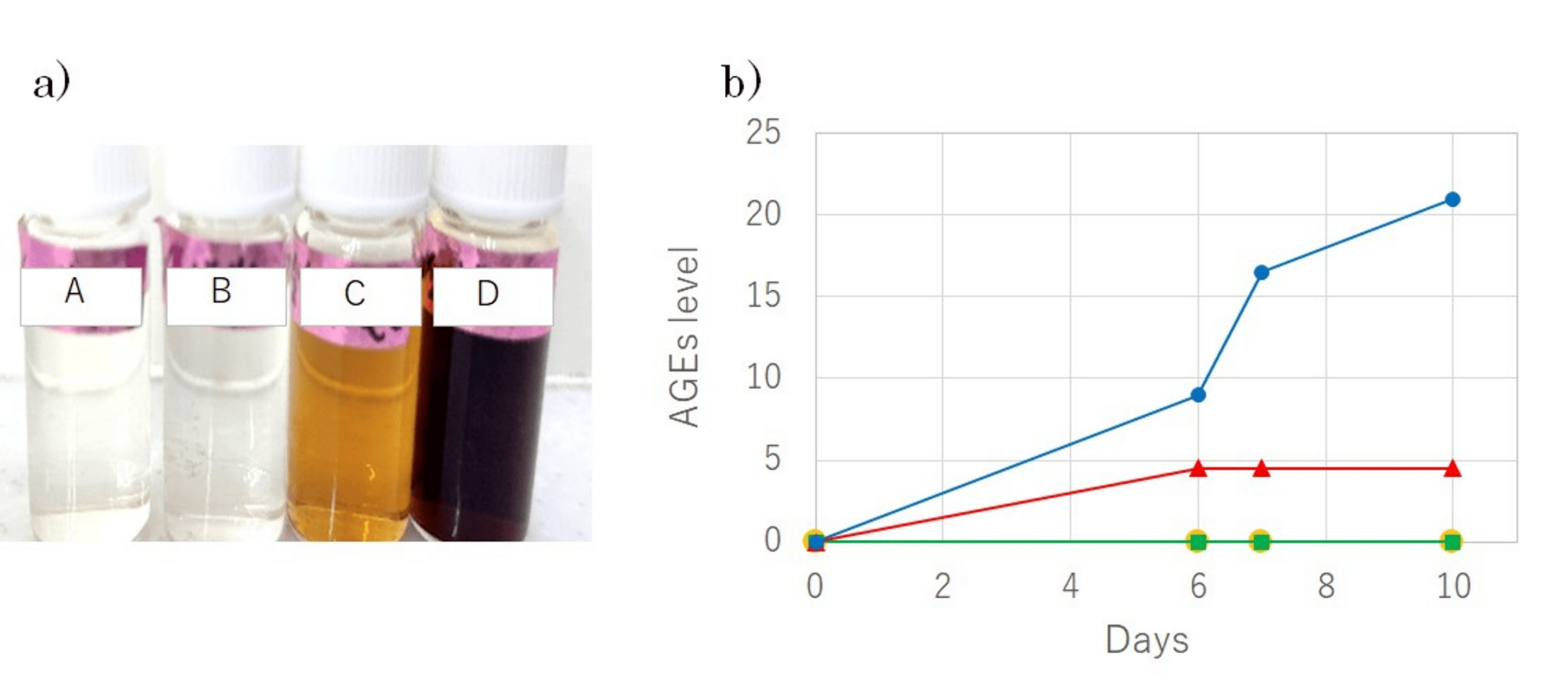
Glycation potential of commercial beverages on collagen (a) Appearance of the tested samples after seven days of glycation: A: unsweetened tap water, B: unsweetened carbonated water, C: fructose-containing water, D: fructose-containing carbonated water; (b) The AGE levels of beverages A, B, C, and D from 0 to 10 days are represented by orange circles, green squares, red triangles, and blue circles, respectively. AGE: advanced glycation end products

Finally, we investigated the extent to which commercially available beverages promote collagen glycation. Neither unsweetened water nor carbonated water exhibited glycation activity. On the other hand, beverages containing an adequate amount of fructose showed collagen glycation activity (Figure 5). Beverage D is sweeter than Beverage C, which is consistent with its higher sugar content (0, 0, 4.8, and 10.4% for Beverages A, B, C, and D, respectively), as measured. Consequently, Beverage D exhibited a stronger glycation effect on collagen. By comparing the results with the fructose concentration-dependent experiments shown in Figure 3, we found that the glycation effect of Beverage D is much greater than that of 10% fructose.

## Discussion

In this study, we developed an AGE colorimetric sheet and applied it to assess collagen glycation under various conditions. Using the sheet, we demonstrated that AGE formation is time-dependent, temperature-dependent, and affected by reducing sugar concentration. In addition, we observed that glycation significantly altered the physical properties of collagen, weakening its gel-forming ability. Furthermore, commercial beverages containing fructose promoted collagen glycation, highlighting the potential role of dietary factors in AGE accumulation. These findings have important implications for public health awareness and education, as well as for advancing research infrastructure related to AGEs.

The observation that fructose induced more extensive glycation than glucose is consistent with previous research, indicating that fructose has a greater glycation potential due to a high proportion of the reactive acyclic form that initially binds to protein amino groups [17,20]. The biochemical properties of fructose are implicated in metabolic diseases, such as insulin resistance and obesity [9,10,16-19]. One of the major AGEs derived from collagen and reducing sugars is pentosidine [6,13,18]. Pentosidine exhibits significant fluorescence and is therefore widely used as a biomarker of AGE accumulation in tissues. The level of pentosidine correlates with the severity of diabetic complications. While further analysis is needed, we believe that this AGE is formed in our assay system, and that this provides an opportunity for young learners to consider the physiological significance of AGE species.

The physical strength of collagen gel was significantly weakened by glycation. Our experiments demonstrated that as the glycation reaction progresses, the collagen gel tends to remain in a sol state instead of forming a solid gel, emphasizing the detrimental effects of glycation on protein structure and function. This observation is consistent with previous findings, which indicate that AGEs induce cross-linking within and between collagen molecules, leading to increased stiffness and reduced elasticity in connective tissues [1,2]. Since collagen is a major structural protein in the human body, its glycation has widespread physiological implications, particularly in aging-related disorders and chronic diseases [16,21,22].

The ability of commercially available beverages to accelerate collagen glycation raises concerns about the role of diet in AGE formation in the body. Indeed, previous studies have shown that excessive consumption of high-fructose foods and beverages leads to increased AGE levels in the human body, which may exacerbate metabolic and inflammatory disorders [17-19]. Our results reinforce the importance of dietary choices in mitigating AGE accumulation, supporting public health efforts to raise awareness of the risks associated with glycation.

Typically, methods for measuring AGEs require specialized analytical instruments and expertise, limiting their accessibility to non-experts and hindering public awareness of AGEs. Our AGE colorimetric sheet bridges this gap by providing a simple and cost-effective method for visualizing and quantifying AGE levels. The potential applications of the innovation in educational settings, healthcare, and community outreach programs will raise awareness of the impact of glycation on health and aging in the general public. Our findings using the practical sheet highlight the impact of dietary components, particularly fructose, on AGE formation and the mechanical properties of collagen. Increased awareness of this risk factor may encourage better lifestyle choices, such as reducing the intake of high-fructose beverages and other AGE-promoting foods, ultimately contributing to healthier aging and the prevention of AGE-related diseases.

The approach developed in this study is beneficial for educational initiatives at various levels. We used commercially available gelatin as a collagen source, along with three different types of sugars: sucrose, glucose, and fructose. A yogurt maker can be used to control the temperature. This approach enables the general public to conduct the experiment without specialized knowledge. Although the experimental accuracy may be lower than when using specialized chemicals and laboratory equipment, a key advantage is that even children can easily and affordably conduct the experiments without the need for specialized equipment [13]. By utilizing the experimental system developed in this study, many individuals will be able to gain a better understanding of AGEs.

The time, dose, and temperature-dependent nature of collagen glycation observed in this study is consistent with previous reports on non-enzymatic glycation processes [23-27]. For example, the Maillard reaction, which leads to AGE formation, is known to advance more rapidly at elevated temperatures [28,29]. These characteristics of the process align with the fundamental principles of chemical reactions in general, providing a clear example of a chemical reaction in scientific education. Therefore, through observations under various experimental conditions, as shown in Figure 3, young learners would comprehend the impact of the type and concentration of reactants, reaction time, and temperature on glycation, leading to an understanding that both the probability of molecular collisions and the affinity between molecules are crucial factors in molecular interactions. Similarly, based on the observations, it becomes easier for non-experts to understand the statement "the amount of AGEs formed in the body is expressed as blood glucose level × duration" or "high-temperature cooking results in a greater formation of AGEs than low-temperature cooking". Furthermore, through our experimental setup using collagen, individuals can gain a deep understanding of the protein, which accounts for 30% of the total protein content in the body and plays essential roles in cartilage, bones, skin, muscles, tendons, and blood vessels [26]. In particular, it will be easy for them to connect the effect of glycation on protein function with aging-related conditions and physical disorders such as wrinkles, age spots, joint pain, vascular diseases, and osteoporosis [3-7]. Therefore, our experimental approach is beneficial at various educational levels.

Moreover, by making this practical colorimetric tool accessible to the general public, including schoolchildren, our study will contribute to the growing body of research on AGEs, support advances in preventive medicine, and promote healthy life expectancy. First, this tool provides an accessible, low-cost method for semi-quantitative analysis of glycation, which may democratize AGE research by enabling its integration into educational settings, citizen science, and resource-limited laboratories. This broader accessibility could accelerate the collection of environmental and dietary data related to AGE formation, supporting large-scale epidemiological studies linking lifestyle habits with AGE accumulation. Second, the colorimetric sheet could serve as a screening tool for identifying high-glycation-potential substances, such as food ingredients, additives, or commercial products. This could inform the development of glycation-inhibiting dietary strategies and functional foods designed to minimize AGE intake and formation, thus aiding research into nutritional prevention of age-related diseases. Third, the demonstrated correlation between glycation level and physical properties of collagen may support future studies exploring the biomechanical consequences of AGE accumulation in tissues. The colorimetric approach may be particularly useful in studies involving tissue engineering, cosmetic science, or orthopedic research, where structural integrity is affected by glycation. Finally, this visual and user-friendly method can be a powerful tool for translational research aimed at public health communication. By fostering awareness of AGEs and their modifiable risk factors, it can bridge the gap between basic biochemical research and preventive medicine, contributing to the global effort to extend healthy life expectancy.

Limitations and future perspectives

Overall, our experimental approach, which bridges academic research and public awareness, is beneficial for educational initiatives and preventive medicine, ultimately promoting healthy aging. Despite its advantages in accessibility and educational utility, our experimental approach has certain limitations. First, the AGE colorimetric sheet provides a qualitative to semi-quantitative assessment of AGE formation based on colorimetric changes. While this method is practical for educational purposes, it lacks the precision and specificity of advanced analytical techniques such as high-performance liquid chromatography (HPLC) or mass spectrometry (MS) [13]. Future studies should aim to refine the sheet’s accuracy by incorporating more precise calibration standards and extending its application to different proteins and saccharides. Second, the method is suitable for measuring water-soluble liquid samples, but further improvements are needed to enable the analysis of oily and solid samples. Third, the colorimetric evaluation may be affected by the visual acuity of individuals, which should be considered and improved in future applications. Fourth, our study primarily focused on collagen glycation under controlled experimental conditions. However, the glycation process is influenced by multiple factors in vivo, including enzymatic activity, oxidative stress, and metabolic pathways [6]. Further research is needed to validate the relevance of our findings in biological systems and assess the applicability of the colorimetric sheet for detecting AGEs in complex biological samples, such as blood or tissue extracts. Additionally, while our study successfully demonstrated the impact of dietary components, particularly fructose, on collagen glycation, the broader implications for long-term dietary habits and AGE accumulation in the body require further investigation. Longitudinal studies exploring the relationship between dietary intake, AGE levels, and health outcomes will provide valuable insights into the preventive potential of dietary modifications [6, 12,30]. Finally, this study highlights the importance of integrating AGE education into public health initiatives. The simplicity and affordability of our experimental system make it a valuable tool for schools, community outreach programs, and healthcare education. Future efforts should focus on developing educational curricula incorporating hands-on experiments using the AGE colorimetric sheet, fostering a deeper understanding of glycation and its role in aging and chronic disease prevention. By addressing these limitations and expanding the scope of our research, we aim to enhance the practical applications of the AGE colorimetric sheet, ultimately contributing to improved public awareness and preventive healthcare strategies.

## Conclusions

Our study demonstrates that a novel AGE colorimetric sheet is an effective and accessible tool for assessing AGE formation in collagen. Our results confirmed that AGE formation is influenced by time, temperature, and sugar concentration, with fructose showing the highest glycation potential. Furthermore, glycation reduced the mechanical strength of collagen. Moreover, fructose-rich commercial beverages accelerated glycation, highlighting the impact of dietary choices on AGE accumulation. The colorimetric tool offers a practical means to understand the impact of AGEs, which may contribute to public health awareness and the development of preventive measures.
